# The mediatory role of challenge and threat in the relationship between positive thinking skills and perception of excellent performance: a study on football players

**DOI:** 10.3389/fpsyg.2025.1513146

**Published:** 2025-04-07

**Authors:** Osman Pepe, Mustafa Can Koc, Cihan Ayhan, Laurentiu-Gabriel Talaghir, Cristina-Corina Bentea

**Affiliations:** ^1^Faculty of Sports Sciences, Süleyman Demirel University, Isparta, Türkiye; ^2^Faculty of Sports Sciences, Istanbul Gelisim University, Istanbul, Türkiye; ^3^Directorate of Sports Sciences Application and Research Center, Istanbul Gelisim University, Istanbul, Türkiye; ^4^Faculty of Sport Science, Sakarya University of Applied Sciences, Sakarya, Türkiye; ^5^Faculty of Physical Education and Sport, Dunarea de Jos University of Galati, Galati, Romania

**Keywords:** football, positive thinking, challenge, threat, excellent performance, perception

## Abstract

**Purpose:**

The present study aims to examine the mediating role of challenge and threat between positive thinking skills and perception of excellent performance in football players competing in the regional amateur league, which is referred to as a semi-professional league in Turkey.

**Methods:**

The study population consisted of football players competing in regional amateur leagues in the 2023–2024 season, and the sample consisted of 388 athletes selected using the simple random method. In addition to the demographic information form developed by the researcher, the Positive Thinking Skills Scale, the Challenge and Threat in Sport Scale and the Performance Perfectionism Scale for Sport were applied to the participants. The data were analyzed digitally through the SPSS 25 package program. The Pearson Correlation analysis was used to determine the correlations between the variables and a regression analysis of the indirect effect approach based on the Bootstrap method through PROCESS v4.2 macro was used to determine the mediating effect of challenge and threat in the relationship between positive thinking and perception of excellent performance. PROCESS Macro Model Option 4 developed by Hayes was used to examine the mediating effect. While conducting this analysis, the 5,000 resampling option was selected in the Bootstrap method.

**Results:**

The study found that positive thinking had a positive, moderate, and statistically significant effect on the perception of challenge (a = 0.439, *p* < 0.01) and excellent performance (c’ = 0.484, *p* < 0.001), with approximately 32% of the variance in challenge explained by positive thinking (*R*^2^ = 0.319, *p* < 0.01). Additionally, challenge played a mediating role. Positive thinking also had a negative, moderate effect on threat (a = −0.425, *p* < 0.01), explaining 7% of the variance in threat (*R*^2^ = 0.070, *p* < 0.01). However, the threat had a negative and low-level impact on the perception of excellent performance (b = −0.244, *p* < 0.001).

**Conclusion:**

In conclusion, it was found that the positive thinking skills of the football players positively affected their perceptions of excellent performance, and the feelings of challenge and threat they experienced on the field played a mediating role in the relationship between these positive thinking skills and perceptions of excellent performance.

## 1 Introduction

Many individuals aspire to achieve excellence in all areas of life, and it is observed that this goal is of particular importance in the field of sports. Especially in highly competitive sport disciplines, athletes aim to rank first in their branches, to win medals, to break records and, most importantly, to be recognized. In order to achieve these aspirations, athletes strive to be perfect with the least amount of mistakes.

Consider specifying what “game of the age” means or provide a reference to support this claim. It would be helpful to clarify whether this phrase refers to football’s historical significance, current popularity, or another aspect. In addition to these skills, athletes must possess physical attributes such as strength, speed, balance, and endurance (Alexe et al., 2024; Samavati Sharif et al., 2024) as well as psychological characteristics including motivation, anxiety, self-confidence, personality, attention, concentration, mental endurance, stress management, and perfectionism (Arısoy and Pepe, 2021; Kalinowski et al., 2020; Watson et al., 2024).

### 1.1 Excellent performance

Perfectionism is a multifaceted characteristic of an individual’s personality which is defined by the presence of excessively high standards coupled with an overly critical evaluation of oneself (Hosseini et al., 2023). While some dimensions and aspects can be seen as positive, benign and adaptive, others can be considered negative, harmful and maladaptive (Chang, 2003; Enns and Cox, 2002). Perfectionism in football is thought to be related to characteristics such as positional competition and skill levels of football players, it can be said that it is related to the personality traits of the football player according to this definition.

Although athletes are expected to perform perfectly in sports, the occurrence of anxiety in the competitive environment may trigger the inhibition of the performance that athletes want to achieve (Flett and Hewitt, 2005). The positive dimension of perfectionism is associated with setting high standards for performance and exhibiting self-control to achieve these standards. On the other hand, the negative dimension of perfectionism is defined as a trait that leads to performance-related errors, threats and hesitancy in behaviors. In this context, it creates a discrepancy between the individual’s expectations and performance outcomes (Bieling et al., 2004; Hill et al., 2004; Suddarth and Slaney, 2001). It should be noted that the competitive environment that occurs in sports can have positive or negative effects on athletes while driving individuals toward excellence.

These positive or negative effects on athletes can occur on athletes’ challenge and threat emotions on the sport fields.

### 1.2 Challenge and threat theory

The literature is in approach that athletes respond to competition in two ways: challenge and threat (Martinek et al., 2003; Seery, 2011). This approach was defined by Jones (1995) in this theory that if athletes react positively to stress in competitive environments, this creates a sense of “challenge” and if they react negatively, this creates a sense of “threat.” Challenge and threat are two different psycho-physiological responses to stressors. A model has been developed to explain the reactions of individuals to such situations, and this model aims to clarify whether the individual is faced with stress or whether they consider stress as a threat to themselves (Meijen et al., 2014). While the feeling of challenge and threat is stated as the emotions that arise only in uncertainty and danger situations, the individual does not experience a feeling of challenge or threat in a situation where there is no danger (Suddarth and Slaney, 2001). Considering how different levels of competition (e.g., amateur and professional) affect feelings of challenge and threat of football players, it is obvious that the physiological and psychological responses required for different levels of competition will differ from each other. It is thought that the feelings of challenge and threat created by different competitive environments (e.g., amateur/professional leagues or national/international cup organizations) in football players may affect their positive thinking skills.

### 1.3 Positive thinking skills

It is known that positive thinking skill is the ability to use the ability to direct our cognitive processes in a positive way.

Conceptually, positive thinking is a way of thinking that does not accept the negatives while accepting that there is something positive that an individual can do in the situations they encounter (İbrahimoğlu, 2024; Willis, 1993). In sports environments, the psychological pressures that athletes are exposed to can negatively affect their performance when they have negative thoughts. However, under these negative conditions, athletes with a positive mindset can overcome negative situations and perform better (Tazegül, 2018).

### 1.4 Present study

The reason for choosing football in this study is that football has evolved from being just a game into a multi-dimensional phenomenon that has created its own industry and values such as sponsorships, broadcasting rights, cultural impact, and global fan engagement. As a major industry, the pursuit of victory both materially and spiritually is central to football.

To date, various psychosocial factors including perfectionism (Koivula et al., 2002; Teixeira et al., 2024; Xu et al., 2024), challenge and threat (Meijen et al., 2020; Öcal and Göncü, 2023; Jones et al., 2009) and positive thinking skills (Çelik et al., 2020; Tazegül, 2018; Şahinler et al., 2020) were studied in different athlete groups. In our literature review, no study was found that examined the relationship between challenge and threat in the relationship between and perception of excellent performance positive thinking skills and perception of excellent performance or challenge and threat in any athlete group.

Additionally, no study has aimed to determine the mediator role of challenge and threat in the relationship between positive thinking skills and perception of excellent performance. It is anticipated that the results obtained from this study will make a significant contribution to the existing literature.

As a preliminary step toward addressing these knowledge gaps, the objectives of this study were to: (a) examine the effect of positive thinking skills on perception of excellent performance and (b) explore the mediation effect of challenge and threat on the relationship between positive thinking skills and perception of excellent performance in football players.

Challenge and threat emotions of football players in competition are thought to play a very important mediating role for positive thinking skills and perceptions of excellent performance.

The present study aims to examine the mediating role of challenge and threat in the relationship between positive thinking and perceptions of excellent performance of football players competing in the regional amateur league, which is referred to as the semi-professional league. For this purpose, the following hypotheses were tested.

*H1*: Positive thinking skills effects on perception of excellent performance.

*H1a*: Challenge and threat has a mediator role on the relationship between positive thinking skills and perception of excellent performance.

## 2 Methods

### 2.1 Research model

**Figure 1:**
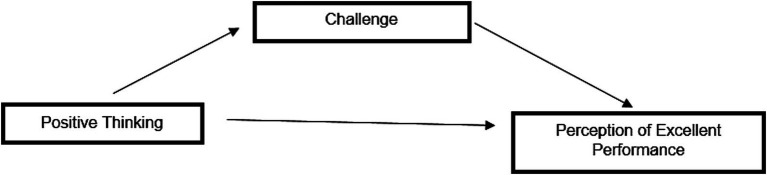
The mediation model of challenge.

**Figure 2:**
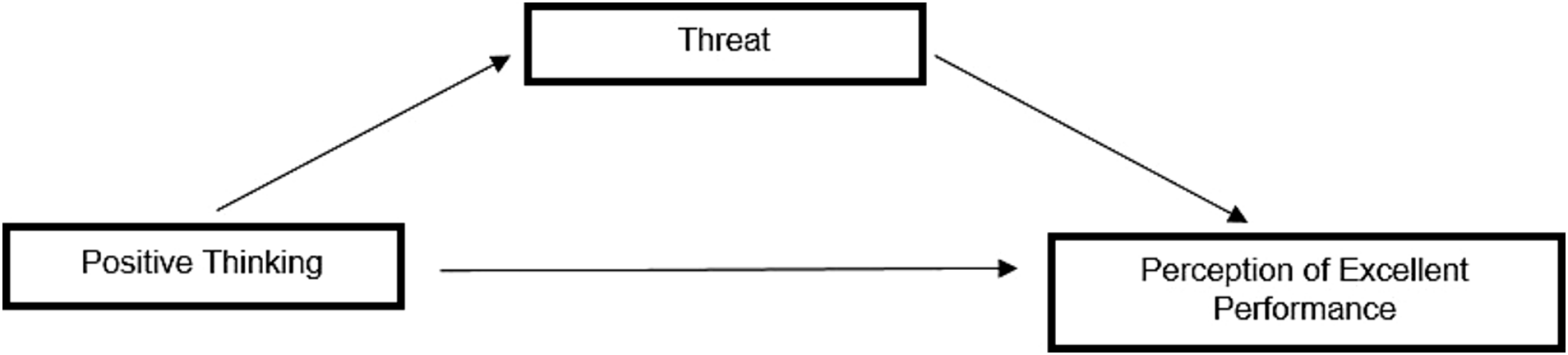
The mediation model of threat.

This study is a correlational survey model study conducted to determine the correlations between positive thinking, challenge, threat and excellent performance perceptions of football players competing in semi-professional regional amateur leagues. A relational survey model aims to identify the presence and extent of relationships between two or more variables (Karasar, 2005). The variables of the present study are positive thinking, challenge, threat and perception of excellent performance. Figures 1, 2 illustrate how challenge and threat mediate the relationship between positive thinking and perception of excellent performance.

When Figure 1 examined, the research model illustrating the mediating relationship among positive thinking, challenge, and perception of excellent performance is presented.

When Figure 2 examined, the research model illustrating the mediating relationship among positive thinking, threat, and perception of excellent performance is presented.

### 2.2 Population and study group

The study population consisted of football players competing in regional amateur league in the 2023–2024 season, and the sample consisted of 388 athletes selected using the simple random method (Çıngı, 1994). When the margin of error was calculated as ±5% in the power analysis, the minimum required sample size was determined to be 384 (Gürbüz and Şahin, 2014). The study utilized a criterion sampling method, where the researcher establishes one or more predetermined criteria to ensure maximum variability (McNabb, 2015). The inclusion criteria were as follows: (i) being older than 18 years of age; (ii) actively playing football in regional amateur league teams during 2023–2024 season in the regional amateur league; and (iii) completing the voluntary informed consent form online. Football players competing in other amateur leagues were excluded from the study. The research was limited to the characteristics addressed, and responses were obtained exclusively from football players who met the inclusion criteria. In addition to the demographic information form developed by the researcher, the Positive Thinking Skills Scale, the Challenge and Threat in Sport Scale and the Performance Perfectionism Scale for Sport were applied to the participants. The necessary ethical and institutional permissions were obtained before the study. All athletes participating in the study fully completed the online questionnaire.

When Table 1 was examined, it was observed that 49.7% of the football players who participated in the study were 18–22 years old, 28.4% were 23–27 years old, 23.7% were 28 years old and older, 22.2% had a sport age of 1–7 years, 37.6% had a sport age of 8–14 years, and 40.2% had a sport age of 15 years or more while 17.8% played in the goalkeeper position, 37.9% played in defense positions, 44.3% played in offense, 37.6% had a professional background and 62.4% did not have a professional background.

**Table 1:** Descriptive statistics of the participants.

Variables	Groups	n	%
Age	18–22	186	49.7
	23–27	110	28.4
	28+	92	23.7
Sport age	1–7	86	22.2
	8–14	146	37.6
	15+	156	40.2
Position	Goalkeeper	69	17.8
	Defense	147	37.9
	Offense	172	44.3
Professional background	Yes	146	37.6
	No	242	62.4

#### 2.2.1 Data collection technique

The study included a personal information form designed by the researcher, consisting of four questions regarding age, years of playing experience, position, professional background, and superstitious beliefs. Data were collected online using Google Forms. The participants started answering the questions after confirming that they voluntarily participated in the study.

##### 2.2.1.1 The Positive Thinking Skills Scale

The Positive Thinking Skills Scale developed by Bekhet and Zauszniewski (2013). The validity and reliability study of the Turkish version of the scale was conducted by Akın et al. (2015). The Positive Thinking Skills Scale is a measurement tool consisting of 8 items. It is scored on a 4-point Likert scale (“0” Never, “1” Rarely, “2” Usually, “3” Always). There is no reverse coded items in the scale. The highest score that can be obtained from the scale is 24 and the lowest score is 0. The Cronbach alpha internal consistency reliability coefficient of the scale was reported as 0.90 (Akın et al., 2015).

##### 2.2.1.2 The Challenge and Threat in Sport Scale

The Challenge and Threat in Sport Scale developed by Rossato et al. (2018). The validity and reliability of the scale for Turkish was conducted by Gürbüz et al. (2021). The Challenge and Threat in Sport Scale is a measurement tool consisting of 11 items and 2 sub-dimensions as “challenge” and “threat.” It is scored on a 5-point Likert scale (“1” strongly disagree, “2” disagree, “3” undecided, “4” agree, “5” strongly agree). The Cronbach’s alpha internal consistency reliability coefficient of the scale was reported as 0.80 for the “challenge” subscale and 0.84 for the “threat” subscale (Gürbüz et al., 2021).

##### 2.2.1.3 The Performance Perfectionism Scale for Sport

The Performance Perfectionism Scale for Sport developed by Hill et al. (2016). The validity and reliability study of the Turkish form of the scale was conducted by Esentaş et al. (2020). The Performance Perfectionism Scale for Sport is a measurement tool consisting of 6 items. It is scored on a 7-point Likert scale. (“1” strongly disagree, “2” disagree, “3” partially disagree, “4” undecided, “5” partially agree, “6” agree, “7” strongly agree). The Spearman-Brown value of the scale was calculated as 0.83 and the Guttman value as 0.80.

#### 2.2.2 Statistical analysis

The statistical analysis of the data to be used in the study was carried out through the SPSS package program. Data normality was assessed by examining skewness and kurtosis values, ensuring they fell within the acceptable range of ±2 (George and Mallery, 2016). As a result of the tests, it was seen that the data showed normal distribution and were found to be suitable for parametric tests. Accordingly, the Pearson Correlation analysis was used to determine the correlations between the variables and a regression analysis of the indirect effect approach based on the Bootstrap method through PROCESS v4.2 macro was used to determine the mediating effect of challenge and threat in the relationship between positive thinking and perception of excellent performance. PROCESS Macro Model Option 4 developed by Hayes (2013) was used to examine the mediating effect. While conducting this analysis, the 5,000 resampling option was selected in the Bootstrap method. The bootstrap method was chosen for its ability to generate reliable confidence intervals without assuming normality in the distribution of indirect effects. Compared to traditional methods like the Sobel test, the bootstrap approach provides more reliable estimates, especially in small to moderate sample sizes, by resampling the data multiple times. This method is widely recommended for mediation analysis due to its improved statistical power and accuracy. The values in the 95% confidence interval obtained in this method are required not to include zero (0) values (Gürbüz, 2019; Hayes, 2013).

## 3 Results

Table 2 shows that the mean positive thinking value of the football players participating in the study was 20.536 ± 4.346, their mean perception of challenge value was 22.070 ± 3.378, their mean perception of threat value was 13.704 ± 7.006 and their mean perception of excellent performance value was 30.083 ± 11.294. It was also found that positive thinking skills had a moderate positive correlation with challenge (*r* = 0.595**, *p* = 0.000), a low negative correlation with threat perception (*r* = −0.264, *p* = 0.000) and a low positive correlation with excellent performance perception (r = 0.258, *p* = 0.000). Challenge perception has a moderate positive correlation with positive thinking skills (*r* = 0.595, *p* = 0.000), a low negative correlation with threat perception (*r* = −0.246, *p* = 0.000) and a low positive correlation with excellent performance perception (*r* = 0.233, *p* = 0.000). Threat perception was found to have a low-level negative correlation with positive thinking skills (*r* = −0.264, *p* = 0.000), a low-level negative correlation with challenge perception (*r* = −0.246, *p* = 0.000) and a low level negative correlation with excellent performance perception (*r* = −0.209, *p* = 0.000). Perception of excellent performance was found to have a low positive correlation with positive thinking skills (*r* = 0.258, *p* = 0.000), a low positive correlation with perception of challenge (*r* = 0.233, *p* = 0.000) and a low negative correlation with perception of threat (*r* = −0.209, *p* = 0.000) (Table 3).

**Table 2:** Descriptive statistics and Pearson correlation coefficients for the correlations between the variables.

	Min	Max	x ± SD	1	2	3	4
1-Positive thinking	7.00	24.00	20.536 ± 4.346	1	0.595**	−0.264**	0.258**
2-Challenge	13.00	25.00	22.070 ± 3.378	0.595**	1	−0.246**	0.233**
3-Threat	6.00	30.00	13.704 ± 7.006	−0.264**	−0.246**	1	−0.209**
4-Excellent performance	13.00	42.00	30.083 ± 11.294	0.258**	0.233**	−0.209**	1

**p < 0.01, *N* = 388.

**Table 3:** The mediating role of challenge between positive thinking skills and perception of excellent performance.

		Result variables				
		Challenge		Perception of excellent performance		
Prediction variables		*b*	*SE*		*b*	*SE*
Positive thinking	a	0.439	0.033	c’	0.484	0.154
Challenge	–	–	–	b	0.426	0.198
Constant	i_M_	13.057	0.686	i_Y_	10.731	3.718
		*R*^2^ = 0.319			*R*^2^ = 0.078	
		*F* = 180.558; *p* < 0.001			*F* = 16.241; *p* < 0.001	

*N* = 388; *p < 0.05, **p < 0.01, ***p < 0.001. SE, Standard error. Unstandardized beta coefficients (b) are reported.

**Figure 3:**
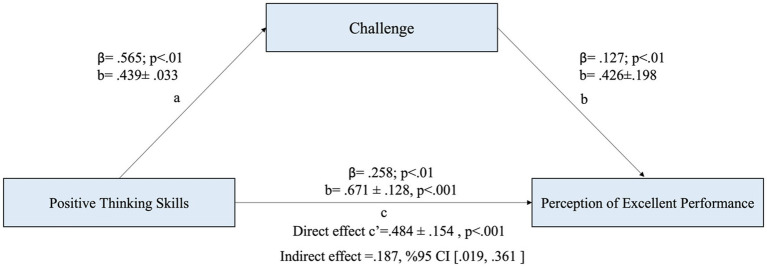
The mediating role of challenge in the relationship between positive thinking and perception of excellent performance.

The results indicate that positive thinking has a moderate and statistically significant effect on challenge (a = 0.439, *p* < 0.01), explaining 32% of the variance in the challenge variable (*R*^2^ = 0.319). This suggests that individuals with higher levels of positive thinking are more likely to perceive challenging situations positively. Additionally, the direct effect of positive thinking on the perception of excellent performance was found to be moderate and statistically significant (c’ = 0.484, *p* < 0.001), indicating that individuals with greater positive thinking skills tend to have a higher perception of excellent performance. Moreover, the challenge variable played a mediating role in this relationship, as it had a positive and statistically significant effect on the perception of excellent performance (b = 0.426, *p* < 0.001). This implies that individuals who perceive challenges positively are more likely to enhance their performance perception. Overall, these findings highlight the importance of positive thinking in fostering a constructive approach to challenges, which in turn contributes to a stronger perception of excellent performance (Figure 3).

Finally, when it was examined whether the challenge variable mediates the relationship between the two variables, the findings show that the indirect effect of the positive thinking variable on the perception of excellent performance is significant, thus the challenge variable mediates the relationship between the two variables (b = 0.426, 95% BCA CI [0.019, 0.361]). As a matter of fact, the bootstrap analysis revealed that the adjusted bias and accelerated confidence interval values (BCA CI) did not include the zero (0) value (Gürbüz, 2019; Hayes, 2013).

**Figure 4:**
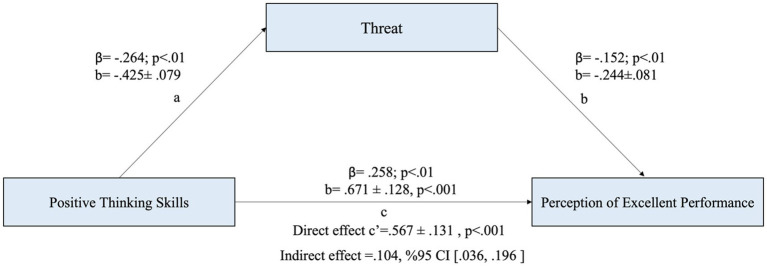
The mediating role of threat in the relationship between positive thinking and perception of excellent performance.

Finally, when it was examined whether the challenge variable mediates the relationship between the two variables, the findings show that the indirect effect of the positive thinking variable on the perception of excellent performance is significant, thus the threat variable mediates the relationship between the two variables (b = −0.425, 95% BCA CI [0.036, 0.196]). As a matter of fact, the bootstrap analysis revealed that the adjusted bias and accelerated confidence interval values (BCA CI) did not include the zero (0) value (Gürbüz, 2019; Hayes, 2013; Table 4; Figure 4).

**Table 4:** Regression analysis results for the study hypotheses and mediation test.

		Result variables				
		Threat		Perception of excellent performance		
Prediction variables		*b*	*SE*		*b*	*SE*
Positive thinking	a	−0.425	0.079	c’	0.567	0.131
Threat	–	–	–	b	−0.244	0.081
Constant	i_M_	22.441	1.661	i_Y_	21.778	3.222
		*R*^2^ = 0.070			*R*^2^ = 0.088	
		*F* = 28.891; *p* < 0.001			*F* = 18.602; *p* < 0.001	

*N* = 388; *p < 0.05, **p < 0.01, ***p < 0.001. SE, Standard error. Unstandardized beta coefficients (b) are reported.

The results indicate that positive thinking had a negative, moderate, and statistically significant effect on threat (a = −0.425, *p* < 0.01), meaning that individuals with higher levels of positive thinking tend to experience lower levels of threat perception. However, only 7% of the variance in the threat variable was explained by positive thinking (*R*^2^ = 0.070), suggesting that other factors also contribute to threat perception. Furthermore, the direct effect of positive thinking on the perception of excellent performance was positive, moderate, and statistically significant (c’ = 0.567, *p* < 0.001). This implies that individuals with higher positive thinking skills are more likely to perceive their performance as excellent. Regarding the mediating role of threat, the results showed that threat had a negative but low-level effect on the perception of excellent performance (b = −0.244, *p* < 0.001). This suggests that while experiencing a sense of threat may slightly reduce performance perception, its impact is relatively weak. Overall, these findings suggest that positive thinking helps reduce threat perception, which in turn has a minor negative effect on performance perception. However, the direct effect of positive thinking on performance perception remains stronger than the indirect effect through threat.

## 4 Discussion

In the present study, it was aimed to examine the mediating role of challenge and threat in the relationship between football players’ positive thinking skills and their perceptions of excellent performance. The model developed in this direction was found to be compatible with the data. Among the variables in the model, it was found that positive thinking skills positively affected the perceptions of excellent performance, in other words, strong positive thinking skills had a significant contribution to the football players’ perceptions of excellent performance. In the literature review, no study examining the effects of positive thinking skills of athlete groups on their perception of excellent performance was found. In the literature, it was reported that there is a positive relationship between positive thinking skills and psychological well-being (Güvenç, 2021; Day and Maltby, 2003), self-efficacy (Alkhatib, 2020), courage (Çelik and Sarıçam, 2018), attitude toward learning (İnce, 2020), life satisfaction (Jung et al., 2007; Leung et al., 2005; Nikmanesh and Mirkazehi, 2020) and interpersonal communication (Karadağ, 2019). There are also studies reporting a positive relationship between the perception of performance and tactical skills (Yıldırım and Göral, 2023), emotion and motivation (Stoeber, 2011), and success goals (Stoeber et al., 2008). Positive thinking is a cognitive process (McGrath, 2004) that helps individuals to have hopes for the future (Akın et al., 2015), develops useful strategies to cope with negativity, and then supports movement toward a positive focus and interpretation (Tod et al., 2011). Athletes with positive thinking skills stand out as individuals who can produce solutions to the problems they face during competitions and perform well by moving away from negative thoughts and focusing on positive thoughts (Gürbüz, 2019). The positive thinking skills of athletes can encourage them to embrace their sports branch, improve their athletic skills, and reach as maximum performance levels (Şahinler et al., 2020). It has been reported that football players have more positive thoughts and exhibit more constructive attitudes toward the problems they face (Tazegül, 2018). In football players, tactics, physical processes and techniques affecting sports performance can show similarities. Therefore, psychological skills are becoming increasingly important for increasing sports performance (Orhan and Ünlü, 2022; Lange-Smith et al., 2024). Sportive performance is considered to be very important for the psychological skills of athletes. It is thought that the inner thoughts and beliefs that the athlete will give himself (sports confidence, positive self-talk, positive thinking) at the point of achieving success will increase his mental endurance and this situation can be reflected in training and/or competition (Grønset et al., 2024; Erdoğan and Kocaekşi, 2015). In this context, the effect of psychological skills alone or in interaction with each other on football players is important. In other words, within the framework of research, in addition to the football players’ high self-confidence and mental endurance levels, the presence of positive thinking skills in the football players can also cause significant increases in the athlete’s performance.

According to the results obtained from the models tested, it was determined that the positive thinking variable of football players had a moderate positive effect on challenge and the challenge variable had a moderate positive effect on the perception of excellent performance, whereas the positive thinking variable of football players had a moderate negative effect on threat and the threat variable had a moderate negative effect on the perception of excellent performance. Therefore, it was found that the feelings of challenge and threat mediated the relationship between the two variables and supported the study hypotheses. However, when the studies in the literature are reviewed, it is seen that there is a limited number of studies on the perception of challenge and threat in sport.

Williams and Cumming (2012) examined the relationship between athletes’ imagery skills and challenge-threat perceptions. As a result of this study, it was determined that imagery was positively related to challenge perception and negatively related to threat perception. In the study conducted by Cankurtaran (2021) with various athlete groups, it was reported that the higher the fear of performance failure of athletes, the higher their perception of threat. Moore et al. (2013) applied challenge and threat manipulations to two groups of experienced golfers during their golf strokes. They reported that the challenge group performed better than the threat group and that it is important to consider the effect of competitive pressure on motor performance. In another study, Türkyılmaz (2019) reported that as the mental endurance of football players increased, the increase in their perception of challenge and the decrease in their perception of threat were statistically related. In general, it is observed that both mental endurance levels and challenge-threat perceptions of athletes are effective in their performances. Theory of challenge and threat situations in athletes suggests that there is a linear relationship between athletes’ competitive stress and struggle and threat. In particular, the theory states that when self-efficacy is high, considering the environment in which competition takes place, athletes perceive the event as challenge; and when self-efficacy is low, they perceive it as threat. While challenge is associated with positive and negative moods, threat is associated only with negative moods (Jones et al., 2009; Yarayan et al., 2022). Within the framework of this information, it is possible to say that the psychological states of athletes are also important in addition to their physical, technical and tactical aspects.

## 5 Results

Finally, it was determined that the positive thinking skills, and perceptions of excellent performance of football players are above average. It was also determined that challenge emotions are above average and threat emotions are under average. it has been determined that positive thinking skills of football players positively affect their perceptions of excellent performance, and the feelings of challenge and threat that they may experience on the field play a mediating role in the relationship between positive thinking skills and perceptions of excellent performance. In line with this information, hypotheses H1 and H1a were accepted.

This is thought to be due to the fact that the football players competing in Turkey’s regional amateur league is generally capable of meeting the psychological and physiological responses required by the league.

## 6 Recommendations

### 6.1 Recommendations for this study

Through this model:

- The Turkish Football Federation, football clubs, and coaches can organize the necessary training to increase psychological levels of football players.
- Coaches can further improve performance by monitoring not only physical abilities but also the players’ psychological levels.
- Additionally, football players can receive support from psychological performance experts to develop their individual psychological profiles.

### 6.2 Recommendations for future studies

- The individual views of participants and their tendency to present themselves in a more favorable light may affect the generalizability of the findings. Therefore, experimental and longitudinal studies similar to the current one should be conducted.
- Studies can be conducted examining the mediator role of challenge and threat feelings in the relationship between positive thinking and perceptions of excellent performance of football players competing in the professional football leagues.
- Additionally, conducting a study like this in different cultural contexts would help determine the impact of cultural factors. By addressing other psychological variables that may affect psychological performance, future research can explore additional relationships mediated by feeling of challenge and threat, thereby advancing our under-standing of athletes’ psychological performance.

## 7 Limitations

This research was conducted exclusively with football players who meet the following criteria:

- Aged at least 18 years old,
- Actively playing football in regional amateur league teams,

Currently participating in the regional amateur league across Turkey in the 2023–2024 season.

Data were collected using the personal information form, the positive thinking skill, the challenge and threat in sport scale, and the performance perfectionism scale for sport. Variables from the Personal Information Form such as age, sport age, position, and professional background were examined in this study.

Furthermore, since the study was designed using a correlational survey model, data were collected within a specific time frame, which limits the ability to establish causal relationships based on the obtained data.

## Data Availability

The datasets presented in this article are not readily available in order to protect the privacy and identities of the participants. Requests to access the datasets should be directed to osmanpepe@sdu.edu.tr.
